# Methods of teaching evidence-based practice: a systematic review

**DOI:** 10.1186/s12909-022-03812-x

**Published:** 2022-10-26

**Authors:** Bethany Howard, Basia Diug, Dragan Ilic

**Affiliations:** Medical Education Research & Quality (MERQ) Unit, School of Public Health & Preventive Medicine, Monash University, Level 1, 553 St Kilda Road, Melbourne, VIC 3004 Australia

**Keywords:** Systematic review, Medical education, Evidence-based practice, Evidence-based medicine

## Abstract

**Background:**

To identify the effectiveness of different teaching modalities on student evidence-based practice (EBP) competency.

**Methods:**

Electronic searches were conducted in MEDLINE, Cochrane central register of controlled trials, PsycINFO, CINAHL, ERIC, A + Education and AEI through to November 2021. We included randomised-controlled trials comparing EBP teaching modes on EBP knowledge, skills, attitudes or behaviour in undergraduate and post-graduate health professions education. Risk of bias was determined using the Cochrane risk of bias tool.

**Results:**

Twenty-one studies were included in the review. Overall, no single teaching modality was identified as being superior to others at significantly increasing learner competency in EBP. Changes in learner knowledge, skills, attitudes and behaviour were conflicting, with studies either reporting no change, or a moderate increase in EBP behavioural outcomes when directly compared to another intervention.

**Conclusion:**

Current evidence highlights the lack of a single teaching modality that is superior than others regarding learner competency in EBP, regardless of health professions discipline or graduate status. The poor quality, heterogeneity of interventions and outcome measures limited conclusions. Further research should focus on the development of high-quality studies and use of psychometrically validated tools to further explore the impact of different EBP teaching modalities.

**Supplementary Information:**

The online version contains supplementary material available at 10.1186/s12909-022-03812-x.

## Background

Evidence-based practice (EBP) is essential for the delivery of quality healthcare [1]. It is a process that allows patients, health professionals, researchers and/or policy makers to make informed health decisions in a given context based on an integration of the best available evidence, with clinical expertise and patient values and preferences [2, 3]. Most commonly this involves five steps: Ask, Acquire, Appraise, Apply and Assess [1, 2, 4]. Competency in EBP is expected by many accreditation bodies, requiring health practitioners to be able to demonstrate skills across the five domains including asking a defined question, literature searching, critical appraisal, integrating evidence into clinical practice and self-reflection [2]. These domains intersect with key EBP competencies of knowledge, skills, attitudes and behaviours – each being critical to the successful implementation of the five steps in clinical practice [2]. However, there still seems to be a gap between the desired and actual practice [5].

Many of the identified barriers to the use and implementation of EBP include those that could be overcome by education [6]. These include inadequate skills and a lack of knowledge particularly pertaining to the research skills required to acquire and appraise studies. In addition to education around the core skills it appears that more practice and exposure to EBP could also overcome barriers, particularly those relating to lack of awareness and negative attitudes [6]. Many practitioners misunderstand EBP as being the ability to keep up to date with research. With an average of over 2000 citations added to Medline each day [7], the ability to effectively and efficiently search and identify relevant, high quality evidence is a critical skill.

A study of undergraduate health student perceptions of the meaning of EBP revealed a very limited understanding of EBP processes or principles [4]. This is despite the fact that over the last two decades EBP has been integrated into core health curricula [2]. The most common teaching methods in undergraduate programs include, research courses and workshops, collaboration with clinical practice, IT technology, assignments, participation in research projects, journal clubs, or embedded librarians [8]. There have been a number of systematic reviews published evaluating the effectiveness of interventions focused on teaching EBP. Some of these have addressed only one aspect of EBP such as literature searching [9], whilst others have focused on specific cohorts such as medical trainees [10], medical students [11], undergraduate health students [12], postgraduate teaching [13], or nursing programs [14].

More recently, MM Bala, T Poklepović Peričić, J Zajac, A Rohwer, J Klugarova, M Välimäki, T Lantta, L Pingani, M Klugar, M Clarke, et al. [15] performed an overview of systematic reviews examining the effects of different teaching approaches for EBP at undergraduate and postgraduate levels. The review identified 22 systematic reviews, with the most recent systematic review published in 2019. This overview of systematic reviews identified that knowledge improved when interventions were compared to no intervention, or pre-test scores [15] across a diverse range of teaching modalities and populations. Similarly, there was positive changes in behaviour, with EBP skills also improving in certain populations. However, of the systematic reviews included only three were judged of high quality, one as moderate, one as low and the other 17 were considered as critically low quality. MM Bala, T Poklepović Peričić, J Zajac, A Rohwer, J Klugarova, M Välimäki, T Lantta, L Pingani, M Klugar, M Clarke, et al. [15] reported that the reasons for categorisation as low-quality were most often a lack of a comprehensive search strategy and/or an adequate risk of bias assessment tool.

As the principles of EBP remain the same irrespective of the health profession, the aim of this systematic review was to identify the current evidence-base on the effectiveness of different teaching modalities on undergraduate or postgraduate learner competency in EBP among all fields of medicine, allied health and health sciences. This review also aims to provide a high-quality update on our 2014 systematic review, which focussed on EBP training in medical trainees. This review expands the population of interest to all health professions trainees at both undergraduate and post-graduate levels, encompassing all areas of EBP competency including knowledge, skills, attitudes and behaviours, including self-efficacy.

## Methods

Cochrane methodology were used to ensure the robustness the systematic approach of this review by following the Cochrane Handbook [16] and reporting in accordance with the PRISMA 2020 statement [17], outlined in the steps below.

### Step 1: Defining the research question and inclusion criteria

A conceptual framework for data synthesis [18, 19], which shares similarities with the EBP PICOTS framework [20], was utilised to determine the inclusion criteria and eligibility of studies to be included in this systematic review (Table 1).

**Table 1:** Conceptual framework for data synthesis

Conceptual framework	PICOTS	Criteria
What works?	Intervention Comparison or Control	Any method of teaching (including lecture, workshops, small group, problem-based, computer assisted, self-directed, or online learning, distance education or bedside teaching) compared with no educational intervention, or a comparison with an intervention listed above
For whom?	Population	Health professions trainees at both undergraduate and post-graduate entry levels
Under which circumstances?	Timing, duration Study type	Randomised-controlled trials with interventions of any duration or frequency
To what end?	Outcome	EBP competency as measured by changes in knowledge, skills, attitudes or behaviour

### Step 2: Searching the literature and the identification of studies

Electronic searches were conducted across the following databases; MEDLINE, Cochrane central register of controlled trials, PsycINFO, CINAHL, ERIC, A + Education and AEI (Informit). No language or date restrictions were imposed. The search was last updated in November 2021. The full search strategy is available in Supplementary file 1. Citations of all articles returned from the search of the respective electronic databases was uploaded for review using *Covidence* [21]. All citations were reviewed independently by two authors (BH and DI) for possible inclusion in the systematic review based on the title and abstract. Full-text of those articles, as well as those where it was not possible to determine inclusion/exclusion based solely on the title and/or abstract was conducted by two authors (BH and DI). Articles that met the selection criteria after final review of the full-text were included in this systematic review. Any discrepancies in author judgement regarding the merits of article selection was resolved by the third author (BD).

### Step 3: Data collection, extraction and management

A data extraction form was piloted before the commencement of data collection and extraction. Two authors (BH and BD) independently extracted data from each included study. Information on the following domains was recorded; study citation, country, setting, study design, study period, inclusion/exclusion criteria, number and type of participants, methodology (including teaching intervention and comparison), outcomes measures and time point, study funding source and conflict of interests. Any discrepancies in author judgement with data extraction were resolved by the third author (DI) before a single consolidated data extraction form was created.

### Step 4: Assessment of risk of bias in included studies

The methodological quality of included studies was assessed using the Cochrane risk of bias tool [22]. Two authors (BH and BD) independently assessed each included study across four domains; 1) random sequence generation (selection bias), 2) allocation concealment (selection bias), 3) blinding of outcome assessment (detection bias) and 4) incomplete outcome data (attrition bias). Risk of bias for each study domain was assessed as ‘high’, ‘unclear’, or ‘low’ risk of bias. Overall risk of bias for the evidence base was similarly assessed. Any discrepancies in author judgement were resolved by the third author (DI).

### Step 5: Data synthesis and analysis

Due to the relative heterogeneity of studies included in this review, a formal meta-analysis was not deemed appropriate. Studies varied across interventions, comparisons, outcomes measured (and tools for measuring outcomes), as well as timing of outcome measurement. Studies also differed with the type of EBP content delivered, with some focussing on single aspects of EBP, whilst others taught all EBP steps as part of the educational intervention. A descriptive analysis was performed on all included studies, with focus on differences in knowledge, skills, behaviour and attitudes between educational interventions and the methodological quality of the evidence.

## Results

### Description of studies

**Fig. 1:**
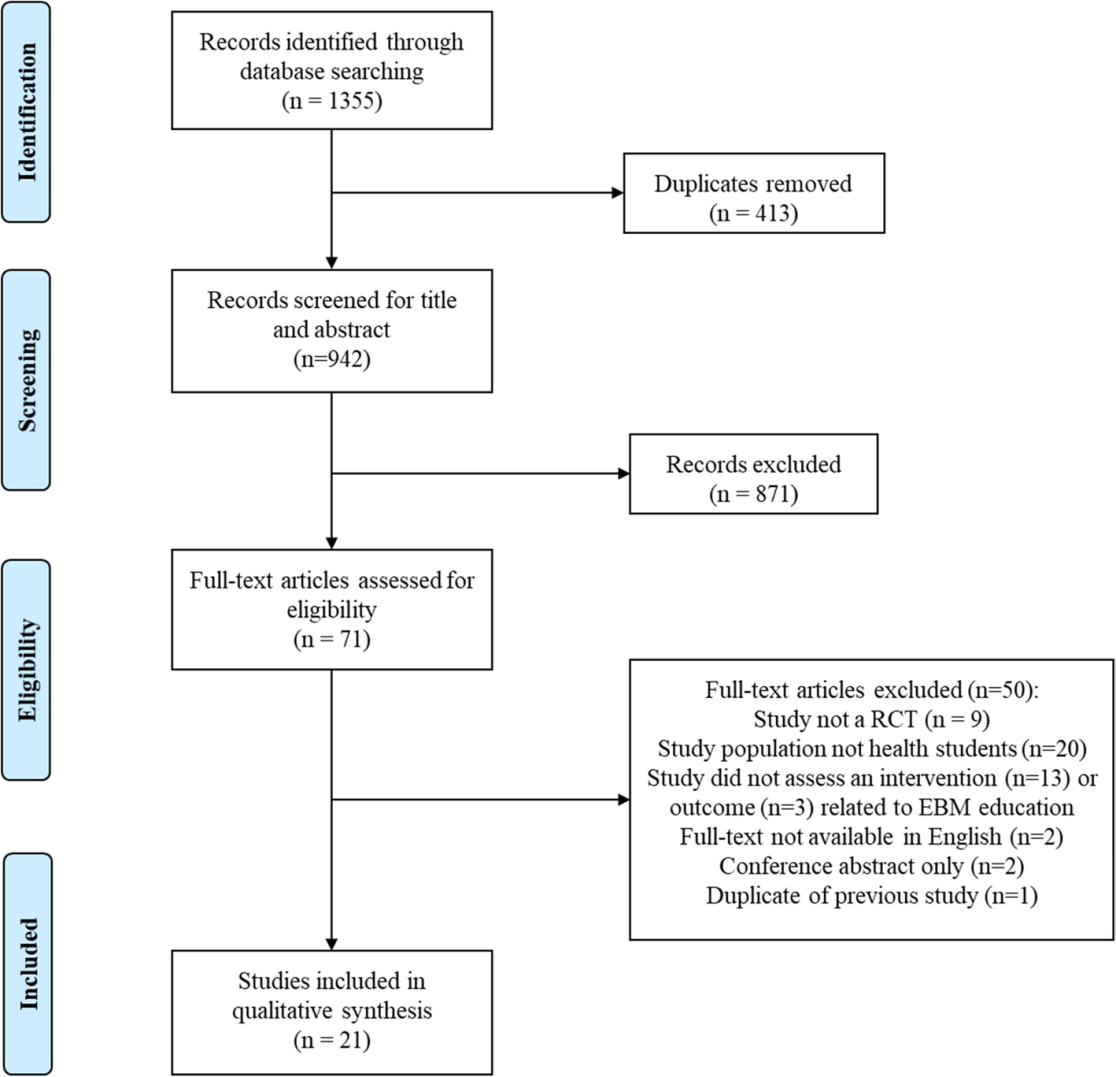
PRISMA flowchart [17]

A total of 1,355 citations were identified from the search, of which 71 were examined for full-text. Twenty-one studies met the inclusion criteria and were included in the review as seen in the PRISMA flowchart (Fig. 1) [17]. Of the 21 studies included in this review, 14 studies were conducted with undergraduate medical students, one with undergraduate osteopathic medical students, one with graduate physician assistant students, one with undergraduate nursing students and one with graduate family nurse practitioner students. Three studies implemented an interdisciplinary approach to teaching with a combination of medical, occupational therapy, physiotherapy, nutrition, pharmacy and dental students. The majority of studies were conducted in the USA, with other studies involved across a variety of countries including Australia, Canada, Hong Kong, Indonesia, Japan, Lebanon, Malaysia, Mexico, Norway, Portugal, Taiwan and United Kingdom. The characteristics of included studies (including information on methodology, participants, interventions, outcomes and findings for each study) are detailed in Table 2.

**Table 2:** Characteristics of included studies

Reference	Methods	Participants	Teaching interventions	Outcomes	Findings
**RG Badgett, JL Paukert and LS Levy ** [23]	Two quasi-randomised controlled studies in the USA	Study 1 (97–98): 159 3^rd^ year undergraduate medical students: • Intervention (*n* = 63) • Control (*n* = 96)	Intervention: 6-h course on how to find and appraise medical evidence with the assistance of a customised computer user interface. The interface included EBM search resources such as Medline, Database of Abstracts of Reviews of Effectiveness (DARE) Control: students who had not yet received instruction	Skills assessed via a non-validated 3-item self-report survey	No difference between groups
		Study 2 (99–00): 151 3^rd^ year undergraduate medical students: • Intervention (*n* = 84) • Control (*n* = 67)	Intervention: 5½-hour course on how to find and appraise evidence using resources accessible via an online portal/website with a focus on SUMsearch. SUMsearch automates searching for medical evidence sourcing the best available evidence Control: students who had not yet received instruction	Skills assessed via a non-validated 4-item self-report survey	No difference between groups Frequency and satisfaction with searching were higher in Study 2
**P Bradley, C Oterholt, J Herrin, L Nordheim and A Bjorndal ** [24]	Randomised controlled trial in Norway	175 10^th^ semester undergraduate medical students: • Directed (*n* = 90) • Self-directed (*n* = 85)	Content, including question framing, literature searching, critical appraisal, application and evaluation of evidence was consistent across both interventions Directed: five 3-h workshops, led by a tutor, covering pre-specified topics Self-directed: a CD, in which the syllabus of the five workshops was outlined, together with teaching materials. Contact with tutors was permitted during the self-directed learning intervention	Knowledge assessed via a non-validated 18-item MCQ tool Attitudes assessed via a 7-item Likert questionnaire based on Taylor et al. [25] Critical appraisal skills assessed via non-validated 60-min exam	No difference between groups
**D Cardoso, F Couto, AF Cardoso, E Bobrowicz-Campos, L Santos, R Rodrigues, V Coutinho, D Pinto, M-A Ramis, MA Rodrigues, et al. ** [26]	Cluster randomised control trial in Portugal	148 8^th^ semester undergraduate nursing students: • Intervention (*n* = 74) • Control (*n* = 74)	Intervention: education as usual plus EBP Education program [27] which addressed models of thinking about EBP, and the types and steps of systematic reviews. Delivery occurred over 17 weeks with three four-hour classroom sessions and three two-hour mentoring sessions with a ratio of one mentor to two–three students Control: education as usual	Knowledge and skills assessed via adapted Fresno tool [28]	Greater improvements in knowledge and skills in the intervention group
**HM Cheng, FR Guo, TF Hsu, SY Chuang, HT Yen, FY Lee, YY Yang, TL Chen, WS Lee, CL Chuang, et al. ** [29]	Randomised controlled trial in Taiwan	94 1^st^ year undergraduate medical students: • Intervention (*n* = 47) • Control (*n* = 47)	All students took part in a 2-week EBM-course during general medicine clinical rotations. Students were also requested to engage in self-directed learning via the provided on-line and e-learning material Intervention: two 1-h structured case conferences addressing the principles and practical applications of EBP Control: no structured case conferences	Knowledge, attitudes, and behaviours assessed by the KAB questionnaire [30]	Higher knowledge and personal application behaviour scores within the intervention group No difference between groups in attitudes or future use behaviours
**J Davis, S Crabb, E Rogers, J Zamora and K Khan ** [31]	Randomised controlled trial in the UK	1^st^ year undergraduate medical students: • Computer-based (*n* = 70) • Lecture-based (*n* = 109)	Teaching duration (40-min) and content was consistent across both interventions including question framing, literature searching, critical appraisal of systematic reviews and meta-analysis and application of findings Computer-based: utilisation of the slides from the lecture-based teaching, with audio over-dubbing and guidance for use on a computer Lecture -based: typical linear lecture format delivered by the same tutor, with opportunities for questions at the end	Knowledge assessed via questionnaires based on the Berlin [32] and Fresno [33] tools Attitudes assessed via a 6-item Likert questionnaire [25]	No difference between groups
**JD Eldredge, DG Bear, SJ Wayne and PP Perea ** [34]	Stratified randomised controlled trial in the USA	71 1^st^ year medical students: • Intervention (*n* = 47) • Control (*n* = 24)	All students received EBM training which consisted of a 1-h lecture, and training labs focusing on searching skills Intervention: students had to assess and provide feedback on their peers’ searches Control: no peer-assessment or feedback	PubMed searching skills assessed via a non-validated test	No difference between groups
**T Hadvani, A Dutta, E Choy, S Kumar, C Molleda, V Parikh, MA Lopez, K Lui, K Ban and SS Wallace ** [35]	Randomised controlled trial in the USA	127127127medical students • Intervention (*n* = 67) • Control (*n* = 60)	All students receive the same EBM curriculum delivered as Intervention: electronic self-paced module with voice over narration and short quizzes throughout the module (approx. 90 min) Control: 60 min traditionally didactic session consisting of a PowerPoint presentation with case examples and discussion delivered by faculty	Critically Appraise Topic (CAT) forms based on Fresno tool [33] Knowledge, attitudes and confidence based on the KACE questionnaire [36]	No difference between groups
**D Ilic, K Tepper and M Misso ** [37]	Randomised controlled trial in Australia	97 3^rd^ year undergraduate medical students: • Intervention (*n* = 60) • Control (*n* = 37)	Intervention: 2-h literature searching skills workshop delivered by a trained subject librarian, which focused on constructing a clinical question and searching the medical literature Control: students were provided with the same workshop upon conclusion of the study	Skills assessed by the Fresno tool [33] and the EBPQ questionnaire [38]	No difference between groups in skills
**D Ilic, RB Nordin, P Glasziou, JK Tilson and E Villanueva ** [39]	Mixed methods randomised controlled trial in Australia and Malaysia	147 3^rd^ year undergraduate medical students: • Intervention (*n* = 73) • Control (*n* = 74)	Intervention: blended learning involving a combination of lecture/tutorial, online and mobile learning. Online components preceded classroom activities and mobile learning occurred on wards Control: classroom teaching only, no online or mobile learning	Attitudes and behaviours assessed via the ACE tool [40] Skills assessed via the Berlin questionnaire [32]	No difference in skills, some differences between groups on specific sub-questions relating to behaviour, attitudes and self-efficacy
**HL Johnson, P Fontelo, CH Olsen, KD Jones, 2nd and RW Gimbel ** [41]	Randomised controlled trial in the USA	35 1^st^ or 2^nd^ year graduate family nurse practitioner students: • Abstracts only (*n* = 19) • Full-texts and abstracts (*n* = 16)	Abstracts only: use of internet accessible search tool (EBN-search) accessed via iPad. Search results provided as abstracts Full-texts and abstracts: search results provided as abstracts and full-text manuscripts	Attitudes assessed via a non-validated Likert questionnaire	No between group differences
**JM Johnston, CM Schooling and GM Leung ** [42]	Randomised controlled crossover ^a^ trial in Hong Kong	129 2^nd^ year undergraduate medical students: • PBL (*n* = 70) • Usual teaching (*n* = 59)	Teaching content was the same in both interventions addressing the steps of EBP: ask, access, appraise, assess, apply Problem based learning (PBL): two 2-h small group PBL case format sessions facilitated by a faculty tutor Usual teaching: one 2-h interactive teaching lesson led by a clinically qualified faculty member and one 2-h small group meeting facilitated by a faculty tutor	Knowledge, attitudes, and behaviours assessed via the KAB questionnaire [30]	Higher attitude scores with usual teaching No difference in knowledge and behaviours between groups
**LA Kloda, JT Boruff and AS Cavalcante ** [43]	Randomised controlled trial in Canada	64 undergraduate occupational or physiotherapy students near the end of their degree: • Alternative clinical question framework (*n* = 30) • PICO framework (*n* = 34)	All students received training about the categories of question types, selection of information resources based on these categories, and advanced search skills in the MEDLINE database in a 90-min session delivered by the same librarian instructor. The only difference between the two groups was the clinical question framework taught and used	Search performance assessed via comparison to gold standard references set. Search skills assessed via a modified Fresno tool [33]	No difference between groups
**D Koufogiannakis, J Buckingham, A Alibhai and D Rayner ** [44]	Randomised controlled trial in Canada	164 1^st^ year undergraduate medical and dental students: • Intervention (*n* = 6 groups) • Control (*n* = 12 groups)	All students had a 3-h lecture encompassing the principles of EBM (i.e. asking an answerable question, levels of evidence and decision making) and took part in weekly 4-h PBL sessions over the course of 6 weeks. Intervention: PBL sessions were attended by a librarian to provide advice to groups Control: PBL sessions without a librarian	Knowledge assessed via a non-validated 10-item MCE tool Attitudes assessed via a non-validated 5-item Likert questionnaire	Higher knowledge scores in the intervention No differences in attitudes between groups
**PM Krueger ** [45]	Randomised controlled trial in the USA	77 3^rd^ year undergraduate osteopathic medical students: • Intervention (*n* = 38) • Control (*n* = 39)	Intervention: 6-h of EBM critical appraisal training consisting of group workshops and lectures Control: no EBM training	Knowledge assessed via non-validated exam questions	Higher exam scores in the intervention group
**GM Leung, JM Johnston, KY Tin, IO Wong, LM Ho, WW Lam and TH Lam ** [46]	Randomised controlled crossover trial in Hong Kong	168 4^th^ year undergraduate medical students: • InfoRetriever (*n* = 54) ^a^ • Pocket card (*n* = 59) ^a^ • Control (*n* = 55) ^a^	Two 2-h interactive sessions addressing the principles and practice of EBM and use of the InfoRetriever or pocket card InfoRetriever: software that provides access to relevant, current and best medical evidence via a personal digital assistant. Students were also provided with a digital version of the pocket card Pocket card: guidelines on clinical decision making and EBM techniques Control: no intervention	Behaviours assessed via the KAB questionnaire [30]	Higher scores for personal and current and future use of EBM with progression from control to pocket card and pocket card to InfoRetriever
**JD Long, P Gannaway, C Ford, R Doumit, N Zeeni, O Sukkarieh-Haraty, A Milane, B Byers, L Harrison, D Hatch, et al. ** [47]	Mixed-method randomised controlled trial in the USA and the Middle-East	68 undergraduate nutrition and 60 PharmD students: • Intervention (*n* = 58) • Control (*n* = 70)	Intervention: use of a web-based evidence-based research (EBR) tool that provided a guide for searching and critical appraisal and links to resources Control: no EBR tool	Skills assessed via a Likert question based on the RRSA [48], and four non-validated questions	Greater improvement in research skills (nutrition students) and ability to distinguish credibility of online sources (PharmD students) in the intervention groups No difference between groups in other measures
**E Nango and Y Tanaka ** [49]	Randomised controlled trial in Japan	17 4^th^, 5^th^, or 6^th^ year undergraduate medical students: • Multidisciplinary (*n* = 7) • Medical-only (*n* = 10)	2-day PBL EBM program addressing topics such as question construction, literature searching, critical appraisal and application Multidisciplinary: medical students participated in groups which included medical, nursing and pharmacy students Medical-only: groups included medical students only	Knowledge assessed vid a non-validated 12-item tool	No difference between groups
**M Sanchez-Mendiola, LF Kieffer-Escobar, S Marin-Beltran, SM Downing and A Schwartz ** [50]	Randomised controlled trial in Mexico	95 5^th^ year undergraduate medical students: • Intervention (*n* = 47) • Control (*n* = 48)	Intervention: 14 two-hour weekly EBM sessions involving large group interactive sessions, small group problem-solving activities, and informatics laboratory sessions Control: students who had not yet received instruction	Knowledge and attitudes assessed via Taylor et al. [25] Knowledge was also assessed via validated a 100-item MCQ test	Higher knowledge and attitude scores and reported use of original research articles and confidence level of critical appraisal skills in the EBM group
**K Schilling, J Wiecha, D Polineni and S Khalil ** [51]	Randomised controlled trial in the USA	237 3^rd^ year undergraduate medical students: • Intervention (*n* = 134) • Control (*n* = 103)	Intervention: students received an online clerkship programme during their 6-week family medicine clerkship. Content addressed the construction of clinical questions, literature searching and appraisal Control: no EBM intervention	Knowledge and skills assessed via a non-validated survey Skills assessed via a case problem	Higher skills scores in the intervention group
**MA Stack, NO DeLellis, W Boeve and RC Satonik ** [52]	Randomised controlled trial in the USA	60 1^st^ year graduate physician assistant students: • Intervention (*n* = 30) • Control (*n* = 30)	Intervention: 16 two-hour weekly EBM sessions involving a variety of teaching techniques (e.g. lectures, small group learning) Control: students who had not yet received instruction	Behaviours assessed via the PECA scale Skills assessed by the Fresno tool [33] and validated EBM self-efficacy scale	Higher knowledge and skills scores and self-efficacy in the intervention group No differences in behaviours between groups
**IS Widyahening, A Findyartini, RW Ranakusuma, E Dewiasty and K Harimurti ** [53]	Randomised crossover trial in Indonesia	220 4^th^ year undergraduate medical students: • Near-peer tutored (*n* = 161) ^a^ • Staff tutored (*n* = 59)^a^	All students took part in a CE-EBM module delivered over 4-weeks involving lectures, plenary presentations and four 2-h tutorials. All tutors completed 3-day teacher training prior to facilitating tutorials Near-peer tutored: newly graduate medical doctor volunteers who had passed the CE-EBM module and practiced using EBM during their clerkship Staff tutored: experienced medical staff who had participated in a 2 or 3-day EBM course and practiced EBM clinically	Knowledge and attitudes assessed based on the Fresno [33] & Berlin [32] tools Skills assessed via the EPIC scale [54]	No differences between groups

^a^Study treated as an RCT with data extracted before crossover

### Methodological quality

**Fig. 2:**
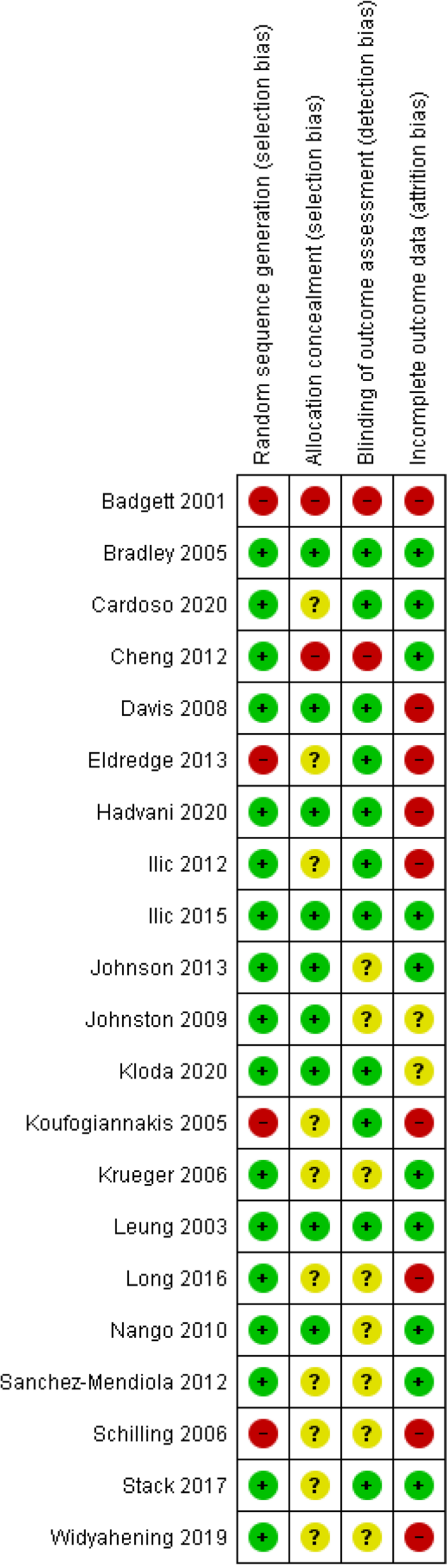
Risk of bias summary. Review authors' judgements about each risk of bias item for each included study

**Fig. 3:**
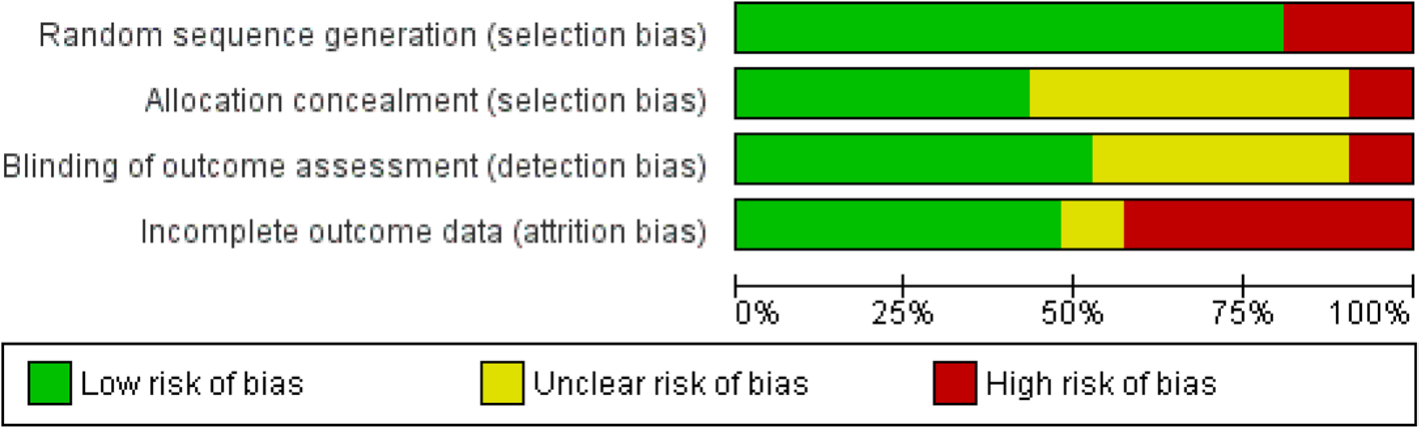
Risk of bias graph. Review authors' judgements about each risk of bias item presented as percentages across all included studies

The risk of bias for each included study is illustrated in Fig. 2. Four studies had a low risk of bias [24, 26, 39, 46], ten studies [23, 29, 31, 34, 35, 37, 44, 47, 51, 53] had a high risk of bias and seven studies [41–43, 45, 49, 50, 52] had uncertain risk of bias as there was a lack of detail in the study methodology to genuinely judge the quality of the study. Table 3 provides further details about the evidence to support judgements about risk of bias for all included studies. Across methodological domains, studies were varied in terms of their risk of bias, with an overall judgement about the summary of evidence being ‘unclear’, in terms of the summary of evidence (Fig. 3).

**Table 3:** Risk of bias overview

	**SELECTION BIAS**		**PERFORMANCE BIAS**	**DETECTION BIAS**	**ATTRITION BIAS**
**Study**	**Random sequence generation**	**Allocation concealment**	**Blinding of participants and personnel**	**Blinding of outcome assessment**	**Incomplete outcome data**
**Badgett 2001** [23]	**High Risk** ‘Group assignment occurred in a quasirandomized manner. At our institution students place themselves into 16 groups of 10 to12 members. The Dean's Office combines these 16 groups into 8 larger groups in order to achieve similar distributions of gender, ethnicity, and academic performance. The Dean's office randomly assigns medical students to a sequence of rotations. A student from each group draws from a bowl a number that indicates the order of clerkship rotations’	**High Risk** Allocation concealment not detailed but it appears as if it was not concealed: 'A student from each group draws from a bowl a number that indicates the order of clerkship rotations'	**High Risk** No blinding	**High Risk** No blinding	**High Risk** Study 1: ‘We excluded 17 incomplete questionnaires.’ Study 2: ‘Of the students with missing results, 24 were on one rotation that we could not access during the survey period. These 24 students had been randomly assigned to a sequence of rotations that placed both their Internal Medicine and Family Medicine rotations in the latter part of the year, and thus the control group contains more missing data.’
**Bradley 2005** [24]	**Low Risk** ‘For each cohort, author CO created an allocation sequence using random number tables’	**Low Risk** ‘Sealed in opaque envelopes, which were ordered numerically before the day of randomisation. Another investigator, PB, completed a list of students meeting the inclusion criteria on the first day of each semester. CO used this list of students and numerically ordered envelopes to sequentially allocate the students to the intervention groups on the same day. The allocation sequence for each cohort was known only to CO until allocation was completed’	**High Risk** ‘Blinding of tutors and students was not possible, but outcome assessors were blinded by use of study codes and examination candidate numbers, known only to CO. The codes were broken only when data analysis was completed.’	**Low Risk** ‘The study was assessed by 2 blinded outcome assessors who could allot 0–5 marks to each of the 4 sections.’	**Low Risk** Many more were lost to follow-up in the directed intervention group for the attitude questionnaire (34 vs 15). Reasons for loss to follow up: Non-attendance at examination, failure to answer question in examination or answer uninterpretable or Non-response despite repeated requests, reason unknown ‘The data were analysed using an intention-to-treat analysis, where participants partially or not receiving the interventions were analysed in the group to which they had originally been randomised. Internal consistency of the knowledge scale ranged from 0.52 to 0.61 across the 3 cohorts with a Cronbach’s alpha of 0.57 for the 3 cohorts combined.’
**Cardoso 2021** [26]	**Low Risk** ‘…students from three clinical nursing courses were randomly assigned to the experimental group (EBP educational program) and students from another three clinical nursing courses were randomly assigned to the control group (no intervention—education as usual) before the baseline assessment. An independent researcher performed this assignment using a random number generator from the random.org website. This assignment was performed based on a list of the 12 optional courses provided through the nursing school’s website.	**Unclear Risk** Not detailed	**High Risk** ‘Due to the nature of the intervention, it was not possible to blind participants regarding treatment assignment nor was it feasible to blind the individuals delivering treatment’	**Low Risk** ‘The rater that graded the answers to the Adapted Fresno Test was blinded to treatment assignment.’ ‘Three independent experts (one psychologist with a doctoral qualification and two qualified nurses, one with a master’s degree) performed a qualitative analysis of the selected monographs. All experts had experience with the EBP approach and were blinded to treatment assignment.’	**Low Risk** Two from the intervention and six from the control group did not fill in the post-test. ‘To minimize the noncompliance impact, an intention-to-treat (ITT) analysis was used to analyze participants in the groups that they were initially randomized to by using the last observation carried forward imputation method.’
**Cheng 2012** [29]	**Low Risk** ‘After rotating schedules were finalised, students were randomly allocated to the above 2 groups using a table of random numbers with even and odd in Group A and B, respectively. A research assistant who was blinded to outcome analysis performed the randomisation as well as allocation of participants.’	**High Risk** ‘Blinding and allocation concealment were not possible in the present study because teachers and students were all aware of the courses they were going to attend.’	**High Risk** ‘Blinding and allocation concealment were not possible in the present study because teachers and students were all aware of the courses they were going to attend. However, study hypothesis had not been disclosed to all participants.’	**High Risk** ‘Blinding and allocation concealment were not possible in the present study because teachers and students were all aware of the courses they were going to attend.’	**Low Risk** Low attrition: ‘A total of 99 undergraduate students rotating to the wards of general medicine were recruited into the EBM teaching courses. Two students were excluded because they didn’t finish the full intervention and complete the post-test. Forty-nine students were randomly allocated to Group A and 48 to Group B. As shown in Fig. 1, 47 subjects in each group completed the post-test questionnaires.’
**Davis 2008** [31]	**Low Risk** ‘Students were randomized to either computer based session or lecture using sealed envelopes prepared by the Birmingham clinical trials unit. The randomization sequence was generated by computer’	**Low Risk** ‘The envelopes were coded by a third party to ensure concealment of randomization.’	**High Risk** Blinding not possible	**Low Risk** ‘The questionnaires were marked by an examiner blind to group allocation.’	**High Risk** Higher amount of missing data for intervention group (44 incomplete ineligible questionnaires compared with 6 in control)
**Eldredge 2013** [34]	**High Risk** ‘The chair of the block (Bear) allocated all students into problem-based learning tutorial groups using their average score on two tests in the previous block. In this way, students were distributed evenly in terms of previous test performance across eleven tutorial groups. The authors used a random number generator to assign each of the eleven tutorial groups into either an intervention or a control group; in other words, randomization was applied at the tutorial group level rather than at the individual level. Scheduling and room size constraints meant that more tutorial groups (seven) were allocated to the intervention group than the control group (four).’	**Unclear Risk** Not detailed. ‘Scheduling and room size constraints meant that more tutorial groups (seven) were allocated to the intervention group than the control group (four).’	**High Risk** Blinding not possible	**Low Risk** ‘The instructor graded all students’ assignments and provided feedback seventy-two hours after the labs using the same three rubrics with all students’ identities concealed.’	**High Risk** Difference in group sizes and number lost in each group is not detailed. ‘During the fall, 80 students began the genetics and neoplasia block. EBM knowledge and skills training constituted a component of this block. Six students were absent during the EBM labs, and 3 did not take the formative test, leaving a total study size of 71.’
**Hadvani 2020** [35]	**Low Risk** ‘We created a computer-generated randomization scheme, where each 2-week block of students were randomized to receive either TDS or SPM	**Low Risk** The investigator (S.K.) created the scheme and was unaware of student rotation assignments. The rotation schedules for students were made by individuals outside the study team from the Undergraduate Medical Education office, who were unaware of the randomization schedule.’	**High Risk** ‘Due to the nature of the intervention, we were unable to blind students and TDS facilitators.’	**Low Risk** ‘Blinding was utilized for assessment of the primary outcome. A single evaluator (A.D.), blinded to the group assignment and student names, graded the forms.’	**High Risk** No reasons given for why post-intervention test results were not completed by participants. Whilst there was minimal drop-outs post-intervention, more than one-third of participants across both groups did not reply to follow-up
**Ilic 2012** [37]	**Low Risk** ‘Participants were randomly assigned independently by the Southern Health clinical site administrator by block randomization to either the intervention or control groups (Fig. 1). A computer random number generator was used to generate a randomization list in blocks of four.’	**Unclear Risk** Not detailed	**High Risk** ‘Blinding of investigators and participants was not possible as the subject librarian and students were aware of their allocation.’	**Low Risk** ‘The outcome assessor and data analyst were blinded to the allocation.’	**High Risk** ‘Data were analyzed using the principle of intention-to-treat.’ However, much more attrition occurred in the control group (22 compared with 2)
**Ilic 2015** [39]	**Low Risk** ‘Students were randomised according to their tutorial group (i.e. cluster) by a researcher independent to the study utilising a simple cluster randomisation procedure (computerised random numbers).’	**Low Risk** ‘Students were randomised according to their tutorial group (i.e. cluster) by a researcher independent to the study utilising a simple cluster randomisation procedure (computerised random numbers).‘	**High Risk** ‘Due to the educational nature of the intervention, it was not possible to blind either the educators or the students.’	**Low Risk** ‘Student competency in EBM was assessed by a blinded outcome assessor. The outcome assessor and data analyst were kept blinded to allocation’	**Low Risk** ‘Quantitative data was analysed using the principle of intention-to-treat.’ ‘A total of 147 (30%) (45 graduate-entry and 102 undergraduate entry) students completed the Berlin Questionnaire and ACE tool (Fig. 1). The remaining 350 students declined to complete the outcome assessment.’
**Johnson 2013** [41]	**Low Risk** ‘This was a simulation-based randomized trial with balanced group allocation [1:1].’	**Low Risk** ‘Following informed consent, study participants drew a sealed envelope from a cardboard box. Inside the envelope was a card with a unique study number and either an “A” or “B” letter associated with group allocation; A = intervention and B = control. In this study intervention group participants (A) were provided access to research abstracts only; control group participants (B) had access to research abstracts and full-text manuscripts.’	**Low Risk** ‘Participants were blinded to their group assignment, as were all co-investigators. Only the principal investigator, who was not associated with the Graduate School of Nursing, had knowledge of individual participation that linked participant name, demographic data, and their questionnaire results.’	**Unclear Risk** It is not clear whether outcome assessors knew which group participants where in	**Low Risk** ‘All participants regardless of allocation completed the exercise and their respective questionnaire; one student did not answer the target question regarding the perceived usefulness of abstract in clinical decision making. In final analysis, this changed the number of participants from 36 to 35 and the intervention group from 20 to 19.’
**Johnston 2009** [42]	**Low Risk** ‘A two-stage randomisation strategy was adopted. First, students were divided into 13 standard learning groups of approximately equal size (9–10 students each) by the Faculty of Medicine's Medical Education Unit, independent of the research team. Seven such learning groups were randomly assigned to the usual teaching intervention arm in the first half of the trial followed by the PBL intervention in the second half and six groups were randomly assigned to the PBL intervention arm followed by usual teaching intervention.’	**Low Risk** ‘Assignment of students and the randomisation process were concealed from both the participants and investigators.’	**High Risk** Blinding not possible (crossover design)	**Unclear Risk** Not detailed in the study	**Unclear Risk** Not fully detailed in the study (shown how many per outcome not per group) ‘KAB response rates were 97% at baseline, 88% at first assessment and 89% at second assessment.’
**Kloda 2020** [43]	**Low Risk** ‘Participants were randomly allocated to the experimental group (the alternative clinical question framework) or the control group (the PICO framework) using an online random number generator after consent was obtained.’	**Low Risk** ‘The allocation was concealed from the students until the instruction session during class time.’	**Low Risk** ‘Both the control and experimental groups were taught by the same librarian instructor who was also one of the researchers (Boruff), for the same ninety-minute duration, and in a face-to-face setting with identical teaching methods (i.e., a combination of lecture and hands-on activities) in a computer-lab classroom setting… The difference between the two sessions amounted to three slides.’	**Low Risk** ‘All participants’ identities were masked and coded so that scores for the information literacy self-efficacy instrument could be compared pre- and post-instruction.’	**Unclear Risk** Study flow diagram is presented, however there is no mention of why participants dropped out, didn't complete, or didn't provide completed/partially completed outcome measures ‘Out of a possible 151 eligible OT and PT students, 103 consented to participate in the study, but several were lost due to withdrawal, drop-out, and failure to follow-up, leaving 64 with data’
**Koufogiannakis 2005** [44]	**High Risk** ‘Students were assigned to groups by the course coordinator before the start of classes, with the aim to have a representative mixture of students (male/female, ethnic background, educational background) in each group. Six librarians participated, all from the John W. Scott Health Sciences Library (one group had two librarians sharing the time equivalent to one librarian—they are counted as one librarian for the purposes of this paper). The librarians were assigned randomly to one of the 18 groups.’	**Unclear Risk** ‘University staff not directly involved with the study blindly drew the librarians’ names from a hat, and a second staff member drew from a separate hat the number of the group to which the librarian would be assigned.’	**High Risk** Blinding not possible	**Low Risk** ‘WebCT allowed individual students to be matched with their scores throughout the research study. This process was managed by the Faculty’s Under-graduate Medical Education office, so that the researchers were kept at an arms length from any information that would identify individual students.’	**High Risk** No information provided on participant numbers for each group or completeness of data
**Krueger 2006** [45]	**Low Risk** ‘The students are assigned by lottery to eight groups, with each group comprising 8 to 10 students. These groups rotate together through the various clinical clerkships The rotating clinical clerkship groups for the 1998–1999 academic year were randomly assigned to a study (EBM) or a control (non-EBM) group. Eight groups of students rotated through the obstetrics and gynecology clerkship that year.’	**Unclear Risk** Not detailed	**High Risk** Blinding not possible	**Unclear Risk** ‘The examination was independently reviewed. Two reviewers with expertise in medical education who were blinded to the study-arm assignment, established face validity. They agreed that the examination assessed critical analysis and the curriculum taught the body of knowledge students would need to demonstrate expertise in critical analysis.’	**Low Risk** ‘Follow-up was available for 100% of the students. All 77 students completed the clerkship and took the examination.’
**Leung 2003** [46]	**Low Risk** ‘The students were randomised in two stages. Firstly, they were randomly divided into three groups of about equal size. Secondly, the groups were randomly allocated to start the clerkship in one of the three teaching blocks (groups A to C).’	**Low Risk** ‘Assignment and randomisation were concealed from the students and investigators’	**High Risk** Blinding not possible	**Low Risk** ‘Investigators had access only to aggregate results and were blinded to data at the individual level’	**Low Risk** ‘Response rates were 100% throughout.’ ‘One student initially randomised to block A (pocket card first) withdrew from medical school and therefore withdrew from medical school and therefore dropped out of the study during their first rotation.’
**Long 2016** [47]	**Low Risk** ‘A table of random numbers was used to allot subjects in each of the RCTs to control or intervention groups.’	**Unclear Risk** Not detailed	**High Risk** Blinding not possible	**Unclear Risk** Not detailed	**High Risk** Sampling was purposefully oversampled to account for attrition. Variance in lost to follow-up between intervention and control groups (nutrition 3 vs 19, pharmacy 9 vs 0). Subjects with missing data for questions 1 or 2 were removed from paired analysis
**Nango 2010** [49]	**Low Risk** ‘Medical students were randomly assigned to either multidisciplinary groups (MultiG) that consisted of six multidisciplinary students (two medical students, two pharmacy students and two nursing students) or medical student groups (MedG) that consisted of six medical students only, in a ratio of 2:3 using computer generated random numbers (Fig. 1). All pharmacy and nursing students were allocated to a MultiG group. Randomization was stratified by gender.’	**Low Risk** ‘Allocation of randomization was concealed until the randomization was completed.’	**High Risk** ‘However, based on study design, it was impossible to blind the nature of the intervention’	**Unclear Risk** Does not appear that outcome assessors were blinded as detail is not provided	**Low Risk** ‘All analyses were performed … according to an intention—to—treat principle.’ ‘One medical student assigned to MultiG and two assigned to MedG did not attend the PBL program, and were excluded from further analysis.’
**Sanchez-Mendiola 2012** [50]	**Low Risk** ‘The randomization was done by the medical school with a computer generated list, using the block randomization method with blocks of two to ensure equal sample sizes’	**Unclear Risk** Not detailed	**High Risk** Blinding not possible	**Unclear Risk** ‘The data analysis was blinded in an attempt to decrease bias.’	**Low Risk** ‘One student from the M5 EBM group was sick on the assessment day.’
**Schilling 2006** [51]	**High Risk** ‘Alternating blocks of clerks were assigned to the intervention group.’	**Unclear Risk** Not detailed	**High Risk** Know if have a librarian or not	**Unclear Risk** Not detailed despite study being called a blinded study	**High Risk** No description of attrition within each group or how analysis accounted for ‘The final EBM case was completed by 85.5% of clerkship students in the blocks that administered the case, with comparable response rates for other outcomes.’
**Stack 2020** [52]	**Low Risk** ‘The sample was randomized into the intervention group and the control (wait) group by simple randomization.’	**Unclear Risk** Not detailed	**High Risk** ‘Participants and course instructors were un-blinded.’	**Low Risk** ‘Outcome measures were scored by trained research assistants who were blinded.’	**Low Risk** ‘One participant dropped out of the control (wait) group due to personal issues before data collection. Thirty students were analysed in each group on an intention to treat basis’ ‘Control group PECA results were analyzed with *n* = 29 as one participant invalided their PECA results by not following protocol. No participants were lost to follow up. Each participant’s data was analyzed in the group to which they were originally randomized (intention to treat principle).’
**Widyahening 2019** [53]	**Low Risk** ‘The students were randomly assigned to groups of 10 or 11. For the first two group discussion sessions, each group was randomly scheduled to be tutored either by medical staff or junior doctors. During the last two group discussion sessions, the groups were crossed over (see Fig. 1). Randomization was implemented using computer software. As medical staff tutors and near-peer tutors were unequal in number, five groups did not participate in the crossover and were tutored by medical staff throughout.’	**Unclear Risk** Not detailed	**High Risk** Blinding not possible	**Unclear Risk** Not detailed	**High Risk** ‘The analysis was based on the intention-to-treat principle’ but there was varying numbers available for different outcomes

### Study population and EBP interventions

Studies differed in the EBP competencies delivered as part of their respective training programs. GM Leung, JM Johnston, KY Tin, IO Wong, LM Ho, WW Lam and TH Lam [46] delivered an introduction to the principles of EBP with undergraduate medical students and D Cardoso, F Couto, AF Cardoso, E Bobrowicz-Campos, L Santos, R Rodrigues, V Coutinho, D Pinto, M-A Ramis, MA Rodrigues, et al. [26] delivered an EBP education program to undergraduate nursing students. The study by PM Krueger [45] focussed on teaching critical appraisal skills to undergraduate osteopathic medical students.

Four studies focussed on teaching searching skills (including constructing a clinical question). Of the four studies, JD Eldredge, DG Bear, SJ Wayne and PP Perea [34] and D Ilic, K Tepper and M Misso [37] training undergraduate medical students, whilst HL Johnson, P Fontelo, CH Olsen, KD Jones, 2nd and RW Gimbel [41] trained graduate family nursing students and LA Kloda, JT Boruff and AS Cavalcante [43] trained undergraduate occupational therapy and physiotherapy students.

Two studies focused on delivering teaching searching and appraising clinical evidence. The study by RG Badgett, JL Paukert and LS Levy [23] consisted of two quasi RCTs with undergraduate medical students, whilst the study by JD Long, P Gannaway, C Ford, R Doumit, N Zeeni, O Sukkarieh-Haraty, A Milane, B Byers, L Harrison, D Hatch, et al. [47] was with pharmacy and nutrition students.

A total of 12 studies delivered teaching programs on the 4 steps of EBP (asking a clinical question, searching the literature, critical appraisal of the evidence, and integration of evidence in the clinical setting). P Bradley, C Oterholt, J Herrin, L Nordheim and A Bjorndal [24], J Davis, S Crabb, E Rogers, J Zamora and K Khan [31], T Hadvani, A Dutta, E Choy, S Kumar, C Molleda, V Parikh, MA Lopez, K Lui, K Ban and SS Wallace [35], D Ilic, RB Nordin, P Glasziou, JK Tilson and E Villanueva [39], JM Johnston, CM Schooling and GM Leung [42], M Sanchez-Mendiola, LF Kieffer-Escobar, S Marin-Beltran, SM Downing and A Schwartz [50] and IS Widyahening, A Findyartini, RW Ranakusuma, E Dewiasty and K Harimurti [53] delivered their programs to undergraduate medical students. HM Cheng, FR Guo, TF Hsu, SY Chuang, HT Yen, FY Lee, YY Yang, TL Chen, WS Lee, CL Chuang, et al. [29] and K Schilling, J Wiecha, D Polineni and S Khalil [51] delivered their programs to undergraduate medical students as an integrated clinical rotation. MA Stack, NO DeLellis, W Boeve and RC Satonik [52] delivered their program to graduate physician assistant students, whilst E Nango and Y Tanaka [49] and D Koufogiannakis, J Buckingham, A Alibhai and D Rayner [44] delivered their programs as part of an interdisciplinary program.

It was noted that none of the studies included long term follow up and assessment of EBP competencies post intervention, with assessment delivered post-intervention.

### EBP competency

#### Knowledge

Twelve studies in total examined the impact of teaching modes on learner knowledge [24, 26, 29, 31, 35, 42, 44, 45, 49–51, 53]. Five studies determined learner knowledge post-intervention via a non-validated tool or survey [24, 44, 45, 49, 51], three utilised either the Berlin or Fresno tool in isolation [26] or combination [31, 53], and another two utilised the Knowledge, Attitude and Behaviours (KAB) questionnaire [29, 42]. Other methods used to determine impacts on learner knowledge included the Knowledge, Attitudes, Access, and Confidence Evaluation (KACE) [35] or Taylor et al. [25] questionnaire [50]. Five of the included studies identified no statistically significant difference in learner knowledge between teaching interventions [24, 31, 35, 42, 49, 53]. Teaching modalities investigated across those five studies included comparisons between directed and self-directed learning; computer versus lecture based; self-directed multimedia vs didactic; PBL versus non-PBL structure; multidisciplinary versus homogenous disciplines; and near peer tutored versus staff tutored session. Two of the included studies identified differences in knowledge scores between teaching interventions. HM Cheng, FR Guo, TF Hsu, SY Chuang, HT Yen, FY Lee, YY Yang, TL Chen, WS Lee, CL Chuang, et al. [29] compared structured case conferencing to lecture based teaching. Learners in the structured case conferences were identified to have significantly higher knowledge scores at follow up (MD = 2.2 95%CI 0.52–3.87). D Koufogiannakis, J Buckingham, A Alibhai and D Rayner [44] identified significantly higher learner knowledge with those that attended EBP-related PBL sessions with a librarian compared to sessions without a librarian. Four studies compared the teaching of an EBM course to no teaching [26, 45, 50, 51]. Unsurprisingly, learners who attended the EBM course had significantly higher knowledge scores when compared to those allocated to the control group.

#### Skills

Twelve studies in total examined the impact of teaching modes on learner EBP skills [23, 24, 26, 34, 35, 37, 39, 43, 47, 51–53]. Impacts on learner EBP skills were assessed after the intervention via the Fresno tool in isolation [26, 35, 43, 52] or in combination with the EBP questionnaire [37], non-validated methods [23, 24, 34, 51], the Berlin tool [39], Research Readiness Self-Assessment (RRSA) [47] or the EBP confidence (EPIC) scale [53]. Eight of the studies concluded no statistically significant difference in learner EBP skill between teaching interventions [23, 24, 34, 37, 39, 43, 53]. Teaching modalities investigated across those eight studies included directed versus self-directed learning; blended versus didactic learning; self-directed multimedia vs didactic; specific workshop on searching versus no workshop; near peer tutored versus staff tutored sessions; and EBP training with and without peer assessment. The studies by MA Stack, NO DeLellis, W Boeve and RC Satonik [52], K Schilling, J Wiecha, D Polineni and S Khalil [51], D Cardoso, F Couto, AF Cardoso, E Bobrowicz-Campos, L Santos, R Rodrigues, V Coutinho, D Pinto, M-A Ramis, MA Rodrigues, et al. [26] and JD Long, P Gannaway, C Ford, R Doumit, N Zeeni, O Sukkarieh-Haraty, A Milane, B Byers, L Harrison, D Hatch, et al. [47] examined the effectiveness of an EBP teaching intervention, or curriculum, to usual practice. MA Stack, NO DeLellis, W Boeve and RC Satonik [52] compared the impact of students undertaking a curriculum with EBM teaching embedded, versus students who undertook a curriculum without EBM content. Students undertaking the EBM-based curriculum demonstrated higher EBM-related skills, and also recorded higher self-efficacy with respect to EBM skills. The study by JD Long, P Gannaway, C Ford, R Doumit, N Zeeni, O Sukkarieh-Haraty, A Milane, B Byers, L Harrison, D Hatch, et al. [47] examined the use of a web-based tool to support student EBM searching and critical appraisal skills. Students reported significantly higher self-efficacy scores when using the EBM-related technology. The study by K Schilling, J Wiecha, D Polineni and S Khalil [51] reported higher EBP skills in students attending an online clerkship in EBP, compared to students that did not. D Cardoso, F Couto, AF Cardoso, E Bobrowicz-Campos, L Santos, R Rodrigues, V Coutinho, D Pinto, M-A Ramis, MA Rodrigues, et al. [26] reported greater improvements in EBP skills for those who participated in the EBP education program.

#### Attitudes

Ten studies in total examined the impact of teaching modes on learner EBP attitudes [24, 31, 35, 39, 41, 42, 44, 50, 53, 55]. The main method for determining impact on attitudes post intervention was via the use of Taylor et al. [25]. Other methods included the use of the KAB questionnaire [29, 42], KACE [35] or Asessing Competency in Evidence based medicine (ACE) [39] tool, or non-validated means. Eight of the included studies identified no significant differences in learner EBP attitudes between teaching interventions [24, 31, 35, 41, 42, 53, 55]. Teaching modalities investigated across those eight studies included directed versus self-directed learning; structured case conference versus lectures; computer-based sessions versus lecture; self-directed multimedia vs didactic; near peer tutoring versus staff tutoring; PBL versus usual teaching; librarian assisted PBL versus non-librarian assisted PBL; and web-based teaching versus usual teaching. The study by D Ilic, RB Nordin, P Glasziou, JK Tilson and E Villanueva [39] examined blended learning versus didactic teaching of EBM. No overall difference in learner attitudes was identified, although several significant differences on sub-questions relating to the tool used to access attitudes was observed. Unsurprisingly, the study by M Sanchez-Mendiola, LF Kieffer-Escobar, S Marin-Beltran, SM Downing and A Schwartz [50] observed significantly higher learner attitudes towards EBP when comparing the implementation of an EBP course to no EBP teaching.

#### Behaviour

Five studies in total examined the impact of teaching modes on learner EBP behaviour [29, 39, 42, 46, 52]. Three studies determined impact on behaviours post intervention by the KAB questionnaire [29, 42, 46], one via the ACE tool [39] and one via the Patient Encounter Clinical Application (PECA) scale [52]. Two of the included studies identified no impact of teaching modes on EBP behaviour (PBL versus usual teaching and EBM curriculum versus curriculum without EBM integration) [42, 52]. The study by D Ilic, RB Nordin, P Glasziou, JK Tilson and E Villanueva [39] identified increases in EBP behaviour sub-scores in learners that received blended learning, versus those that received a didactic approach. HM Cheng, FR Guo, TF Hsu, SY Chuang, HT Yen, FY Lee, YY Yang, TL Chen, WS Lee, CL Chuang, et al. [29] reported increases in EBP behaviour in learners that were exposed to case conference style teaching of EBP, compared to those that received lecture-based sessions. Unsurprisingly, students who received any form of EBP teaching reported higher EBP behavioural scores compared to students that weren’t exposed to any form of EBP teaching [46].

## Discussion

The findings of this systematic review build on the emerging evidence base exploring the effectiveness of different teaching strategies on the competency of EBP learners [9–15]. Results from this current review update and extend on the findings from our 2014 systematic review, which identified a small, albeit moderate quality of evidence, on the effectiveness of different training modalities in medical trainees [10]. Although our current study expanded the search to include allied and health sciences trainees, very few additional studies across these health professions have been performed. As per our 2014 review, our current findings highlight the variability in methodology quality, and use of psychometrically validated tools to assess learner competency in EBP [10]. These results align with the most recent overview of systematic reviews, which concluded the current limitations of evidence on the topic to be poor quality, heterogeneity of interventions and outcome measures [15].

In a bid for EBP education to be more ‘evidence-based’, a 2018 consensus statement was developed detailing the minimum core competencies in EBP that health professionals should meet to improve and standardise education in the discipline [2]. For such competencies to be translated into practice, a variety of robust teaching implementation and assessment strategies and tools must be available. A systematic review of evaluation tools in 2006 identified over 104 assessment instruments, of which only two were psychometrically evaluated [56]. The CREATE framework was developed in 2011, with the objective of creating a common taxonomy for assessment tools to cover assessing all steps of competency in EBP (including asking; acquiring; appraising; applying and assessing) [57]. A 2020 extension of the earlier 2006 systematic review identified six tools of reasonable validity evaluating some, but not all aspects of EBP [58].

Whilst our systematic review included 21 studies for review, the heterogeneity between the studies in terms of how outcomes were measured precluded any meaningful meta-analysis. I Chalmers and P Glasziou [59] have highlighted the impact of research waste in medical literature. Therefore, future research in the EBP education field must avoid waste by assessing agreed-upon outcome measures with robust, psychometrically validated tools. Such assessment tools should be competency focussed, rather than discipline focused, with the emphasis on evaluating specific EBP domains as recommended by the CREATE framework [40, 57]. Use of anything else only contributes to the gathering pile of research waste.

The findings from this systematic review suggest that insufficient evidence to promote one teaching modality over another in terms of its effectiveness on EBP learner outcomes. What is common across the current evidence is the need for multi-faceted, interactive, authentic learning experiences and assessment. Teaching utilities such as journal clubs have the potential to incorporate strong andragogic principles, for example with the use of PBL principles, coupled with a teaching modality that is commonly used in practice as a method of professional education. Further research is required to better understand how such authentic teaching and learning utilities are best served in the novice to expert continuum. For example, should novice EBP competencies be scaffolded through structured teaching modalities such as PBL, whilst those with a higher level of competency engage in more ‘authentic’ utilities such as journal clubs?

An important aspect of education not included in any study to date is the impact of cost and value on the teaching and learning experience [60]. The *Prato Statement*, published in 2017, highlights the goal of incorporating economic analyses into health professions education research in order to create an evidence that maximises value – both from an educational and economic perspective [60]. Whilst findings from our review demonstrate relevant equivalence between teaching modalities with respect to measured EBP competencies, the availability of economic data could well provide evidence to persuade the ‘value’ of one teaching modality over another.

Van der Vleuten’s assessment utility formula incorporates cost as a key quality characteristic of assessment [61]. Cost and value are important ingredients that educators should consider when evaluating the effectiveness of teaching strategies, from both a pragmatic and academic perspectives. The number of studies incorporating some form of economic evaluation is growing within the health professions education, however the quality of their reporting is poor [62]. The use of reporting guidelines to incorporate well-constructed economic evaluations as part of the assessment outcomes, particularly in the growing evidence base of EBP teaching, would provide sufficient evidence to conduct sensitivity analyses and provide a different lens from which to interpret evidence – particularly when it seems inconclusive on face value.

Many of the studies included in this systematic review were assessed as having either a high, or unclear, risk of bias. This potential for methodological bias brings a certain level of uncertainty in the evidence base with respect to interpreting the overall results of the review. A further limitation was the heterogeneity between studies with respect to outcomes measured, and tools used to measure these end-points. This variance in outcome measures prevented the possibility of conducting a meta-analysis, which would bring some level of quantifiable assessment of study outcomes. Subsequently, it was not possible to conduct a funnel plot analysis of studies and the potential impact of publication bias on this systematic review. Similarly, the included studies provided little information regarding the educational interventions that could allow reproducibility of the educational method, thereby adding to the ‘educational’ heterogeneity of the studies. All included studies focussed assessment of EBP competency immediately after intervention. The lack of long term follow-up is a significant evidence gap, as it critical questions regarding the need, and frequency, of continued professional development in EBP remain unanswered – particularly the impact that time, resources and environment may have upon self-efficacy, behaviours and attitudes toward implementing EBP principles in practice.

The majority of studies to date have focussed on medical trainees. Further research is required, particularly in the development of high-quality methodological studies to explore the impact of different teaching modalities across the broad spectrum of health professions disciplines. Such studies should focus on assessing key EBP competencies across the domains of knowledge, skills, attitudes and behaviours using robust, psychometrically validated outcome assessment tools.

## Conclusions

The current evidence suggests limited differences on learner competency in EBP across different teaching modalities. Future studies should focus on conducting high methodological studies, with specific focus on measuring core EBP competencies using validated tools across disciplines within the health professions. Similarly, future studies should explore the use of emerging teaching strategies, and their effectiveness in teaching EBP across different stages of training. The COVID-19 pandemic has seen the need for many educational programs to pivot to an online delivery, with many adopting a hybrid online/face-to-face engagement as pandemic restrictions ease. Future work is needed to identify how successfully teaching of EBP can be translated into these emerging teaching modalities. There is also a need to explore long term follow up of learner competency in EBP as learners move along the novice to expert continuum from students to clinicians practicing EBP in the clinical environment.

## Supplementary Information

**Additional file 1.**

## Data Availability

All data generated during this study are included in this published article.
