# Supplementary material for: Methods of teaching evidence-based practice: a systematic review
Source: BMC Med Educ. 2022 Oct 26;22:742. doi: 10.1186/s12909-022-03812-x (PMC9607697; doi:10.1186/s12909-022-03812-x)
Supplement: Supplementary file 1 — Additional file 1. [file 12909_2022_3812_MOESM1_ESM.pdf]

Supplementary file 1 – Search strategy

Search Terms

	**P: Health Students**	**I & C:**	**O: Evidence based practice**
MEDLINE & Cochrane<br>MeSH terms	students, health occupations (exp)<br>education, medical (exp)<br>education, premedical<br>education, nursing (exp)<br>education, pharmacy (exp)<br>education, dental (exp)<br>education, predental<br>education, public health professional	curriculum<br>teaching (exp)<br>lecture<br>computer-assisted instruction<br>education, distance	evidence-based practice (exp)<br>decision making<br>problem solving
PsycINFO<br>MeSH terms	medical students<br>nursing students<br>dental students<br>medical education<br>nursing education<br>psychology education<br>graduate psychology education	curriculum<br>teaching methods (exp)	evidence based practice<br>decision making<br>problem solving<br>critical thinking
CINAHL	students, health occupations (exp)<br>education, health sciences (exp)	curriculum<br>teaching methods (exp)<br>learning methods (exp)	professional practice, evidence-based (exp)<br>decision making<br>problem solving<br>critical thinking
ERIC Thesaurus<br>MAINSUBJECT.EXACT(term)	allied health occupations education<br>clinical teaching (health professions)	teaching methods (exp<br>learning activities (exp)<br>group instruction (exp)<br>computer assisted instruction	evidence based practice<br>decision making<br>decision making skills<br>problem solving

		distance education<br>problem based learning<br>electronic learning<br>active learning<br>workshops	critical analysis<br>critical thinking
A+ & AEI Thesaurus<br>SUA="term"	allied health occupations education<br>medical education<br>graduate medical education<br>medical students<br>graduate medical students<br>health sciences	learning activities<br>lecture method<br>computer assisted teaching<br>distance education<br>problem based learning<br>case studies<br>small group teaching<br>workshops<br>online learning<br>independent study<br>self directed groups<br>blended learning<br>direct instruction<br>experimental teaching	evidence based practice<br>decision making<br>decision making skills<br>problem solving<br>critical thinking
Keyword terms (all)	student* ADJ2 (health OR clinical OR clinician* OR medic* OR doctor* OR physician* OR nurse OR nursing OR pharmac* OR psycholog* OR dental OR dentist* OR midwi#e* OR physiotherap* OR physical therap* OR occupational therap* OR paramedic* OR radiotherapy OR radiation OR podiat* OR optometr* OR speech therap* OR speech patholog* OR dietetic OR dieti#ian* OR nutrition OR exercise physiolog* OR exercise science* OR chiropract* OR biomedic*)<br>education ADJ2 (health profession* OR	curricul*<br>teaching<br>learning<br>lecture*<br>computer-assisted instruction<br>distance education<br>PBL<br>CBL<br>case stud*<br>workshop*<br>e-learning OR elearning<br>independent study OR independent studies<br>direct instruction*	evidence-based practice* OR EBP OR EBPC<br>evidence-based medicine OR EBM<br>evidence-based nursing OR EBN<br>evidence-based healthcare OR EBHC<br>evidence-based dentistry<br>evidence-based decision*<br>evidence-informed practice<br>best ADJ2 evidence<br>PICO<br>clinical question*<br>literature search*<br>critical appraisal<br>critical analys*

	allied health OR health occupation* OR health science* OR medic* OR clinical OR nurse OR nursing OR pharmacy OR psychology OR dental OR dentist* OR midwi#e* OR physiotherap* OR physical therap* OR occupational therap* OR paramedic* OR radiotherap* OR radiation OR podiatr* OR optometr* OR dietetic* OR dieti#ian* OR speech therap* OR speech patholog* OR chiropract* OR biomedic* OR public health)		critical think*<br>decision making<br>problem solving

OVID Medline & Cochrane search strategy

	SEARCH TERMS	MedLine	Cochrane
1 (P)	exp Students, Health Occupations/ OR exp Education, Medical/ OR Education, Premedical/ OR exp Education, Nursing/ OR exp Education, Pharmacy/ OR exp Education, Dental/ OR Education, Predental/ OR Education, Public Health Professional/ OR student* ADJ2 (health OR clinical OR clinician* OR medic* OR doctor* OR physician* OR nurse OR nursing OR pharmac* OR psycholog* OR dental OR dentist* OR midwi#e* OR physiotherap* OR physical therap* OR occupational therap* OR paramedic* OR radiotherapy OR radiation OR podiat* OR optometr* OR speech therap* OR speech patholog* OR dietetic OR dieti#ian* OR nutrition OR exercise physiolog* OR exercise science* OR chiropract* OR biomedic*) OR education ADJ2 (health profession* OR allied health OR health occupation* OR health science* OR medic* OR clinical OR nurse OR nursing OR pharmacy OR psychology OR dental OR dentist* OR midwi#e* OR physiotherap* OR physical therap* OR occupational therap* OR paramedic* OR radiotherap* OR radiation OR podiatr* OR optometr* OR dietetic* OR dieti#ian* OR speech therap* OR speech patholog* OR chiropract* OR biomedic* OR public health)	331937	14266
2 (1 & C)	Curriculum/ OR exp Teaching/ OR Lecture/ OR Computer-Assisted Instruction/ OR Education, Distance/ OR curricul* OR teaching OR learning OR lecture* OR computer-assisted instruction OR distance education OR PBL OR CBL OR case stud* OR workshop* OR e-learning OR elearning OR independent study OR independent studies OR direct instruction*	622418	42961
3 (O)	exp Evidence-Based Practice/ OR evidence-based practice* OR EBP OR EBPC OR evidence-based medicine OR EBM OR evidence-based nursing OR EBN OR evidence-based healthcare OR EBHC OR evidence-based dentistry OR evidence-based decision* OR evidence-informed practice OR best ADJ2 evidence OR Decision Making/ OR Problem Solving/ OR PICO OR clinical question* OR literature search* OR critical appraisal OR critical analys* OR critical think* OR decision making OR problem solving	340810	27774
4	1 AND 2 AND 3	9997	652
5	control* trial*	653898	481936
6	4 AND 5	464	313

OVID PsycINFO search strategy

	SEARCH TERMS	N
1 (P)	Medical Student/ OR Nursing Students/ OR Dental Students/ OR Medical Education/ OR Nursing Education/ OR Psychology Education/ OR Graduate Psychology Education/ OR student* ADJ2 (health OR clinical OR clinician* OR medic* OR doctor* OR physician* OR nurse OR nursing OR pharmac* OR psycholog* OR dental OR dentist* OR midwi#e* OR physiotherap* OR physical therap* OR occupational therap* OR paramedic* OR radiotherapy OR radiation OR podiat* OR optometr* OR speech therap* OR speech patholog* OR dietetic OR dieti#ian* OR nutrition OR exercise physiolog* OR exercise science* OR chiropract* OR biomedic*) OR education ADJ2 (health profession* OR allied health OR health occupation* OR health science* OR medic* OR clinical OR nurse OR nursing OR pharmacy OR psychology OR dental OR dentist* OR midwi#e* OR physiotherap* OR physical therap* OR occupational therap* OR paramedic* OR radiotherap* OR radiation OR podiatr* OR optometr* OR dietetic* OR dieti#ian* OR speech therap* OR speech patholog* OR chiropract* OR biomedic* OR public health)	77352
2 (1 & C)	Curriculum/ OR exp teaching methods/ OR curricul* OR teaching OR learning OR lecture* OR computer-assisted instruction OR distance education OR PBL OR CBL OR case stud* OR workshop* OR e-learning OR elearning OR independent study OR independent studies OR direct instruction*	674957
3 (O)	evidence based practice/ OR evidence-based practice* OR EBP OR EBPC OR evidence-based medicine OR EBM OR evidence-based nursing OR EBN OR evidence-based healthcare OR EBHC OR evidence-based dentistry OR evidence-based decision* OR evidence-informed practice OR best ADJ2 evidence OR Decision Making/ OR Problem Solving/ OR Critical Thinking/ OR PICO OR clinical question* OR literature search* OR critical appraisal OR critical analys* OR critical think* OR decision making OR problem solving	199120
4	1 AND 2 AND 3	3273
5	control* trial*	37996
6	4 AND 5	54

CINAHL search strategy

	SEARCH TERMS	N
1 (P)	(MH "Students, Health Occupations+") OR (MH "Education, Health Sciences+") OR (student* N2 (health OR clinical OR clinician* OR medic* OR doctor* OR physician* OR nurse OR nursing OR pharmac* OR psycholog* OR dental OR dentist* OR midwi#e* OR physiotherap* OR "physical therap*" OR "occupational therap*" OR paramedic* OR radiotherapy OR radiation OR podiat* OR optometr* OR "speech therap*" OR "speech patholog*" OR dietetic OR dieti#ian* OR nutrition OR "exercise physiolog*" OR "exercise science*" OR chiropract* OR biomedic*)) OR (education N2 ("health profession*" OR "allied health" OR "health occupation*" OR "health science*" OR medic* OR clinical OR nurse OR nursing OR pharmacy OR psychology OR dental OR dentist* OR midwi#e* OR physiotherap* OR "physical therap*" OR "occupational therap*" OR paramedic* OR radiotherap* OR radiation OR podiatr* OR optometr* OR dietetic* OR dieti#ian* OR "speech therap*" OR "speech patholog*" OR chiropract* OR biomedic* OR "public health"))	343176
2 (I&C)	(MH "Curriculum") OR (MH "Teaching Methods+") OR curricul* OR teaching OR learning OR lecture* OR "computer-assisted instruction" OR "distance education" OR PBL OR CBL OR "case stud*" OR workshop* OR e-learning OR elearning OR "independent study" OR "independent studies" OR "direct instruction*"	337667
3 (O)	(MH "Professional Practice, Evidence-Based+") OR "evidence-based practice*" OR EBP OR EBPC OR "evidence-based medicine" OR EBM OR "evidence-based nursing" OR EBN OR "evidence-based healthcare" OR EBHC OR "evidence-based dentistry" OR "evidence-based decision*" OR "evidence-informed practice" OR best N2 evidence OR (MH "Decision Making") OR (MH "Problem Solving") OR (MH "Critical Thinking") OR PICO OR "clinical question*" OR "literature search*" OR "critical appraisal" OR "critical analys*" OR "critical think*" OR "decision making" OR "problem solving"	233279
4	1 AND 2 AND 3	10448
5	"control* trial*"	146858
6	4 AND 5	211

ERIC (Proquest) search strategy

	SEARCH TERMS	N
1 (P)	MAINSUBJECT.EXACT("Allied Health Occupations Education") OR MAINSUBJECT.EXACT("Clinical Teaching (Health Professions)") OR noft(student* NEAR/2 (health OR clinic* OR medic* OR doctor* OR physi* OR nurs* OR pharmac* OR psycholog* OR dent* OR midwi?e* OR "occupational therap*" OR paramedic* OR radi* OR podiat* OR optometr* OR speech OR diet* OR nutrition OR exercise OR chiropract* OR biomedic*))OR noft(education NEAR/2 (health OR medic* OR clinical OR nurs* OR pharmacy OR psychology OR dent* OR midwi?e* OR physi* OR "occupational therap*" OR paramedic* OR radi* OR podiatr* OR optometr* OR diet* OR speech OR chiropract* OR biomedic*))	87783
2 (I&C)	MAINSUBJECT.EXACT.EXPLODE("Teaching Methods") OR MAINSUBJECT.EXACT.EXPLODE("Learning Activities") OR MAINSUBJECT.EXACT.EXPLODE("Group Instruction") OR MAINSUBJECT.EXACT("Computer Assisted Instruction") OR MAINSUBJECT.EXACT("Distance Education") OR MAINSUBJECT.EXACT("Problem Based Learning") OR MAINSUBJECT.EXACT("Electronic Learning") OR MAINSUBJECT.EXACT("Active Learning") OR MAINSUBJECT.EXACT("Workshops") OR curricul* OR teaching OR learning OR lecture* OR "computer-assisted instruction" OR "distance education" OR PBL OR CBL OR "case stud*" OR workshop* OR e-learning OR elearning OR "independent study" OR "independent studies" OR "direct instruction*"	832630
3 (O)	MAINSUBJECT.EXACT("Evidence Based Practice") OR "evidence-based practice*" OR EBP OR EBPC OR "evidence-based medicine" OR EBM OR "evidence-based nursing" OR EBN OR "evidence-based healthcare" OR EBHC OR "evidence-based dentistry" OR "evidence-based decision*" OR "evidence-informed practice" OR best NEAR/2 evidence OR MAINSUBJECT.EXACT("Decision Making") OR MAINSUBJECT.EXACT("Decision Making Skills") OR MAINSUBJECT.EXACT("Problem Solving") OR MAINSUBJECT.EXACT("Critical Analysis") OR MAINSUBJECT.EXACT("Critical Thinking") OR PICO OR "clinical question*" OR "literature search*" OR critical OR "decision making" OR "problem solving"	175497
4	1 AND 2 AND 3	6065
5	noft("control* trial*")	3099
6	4 AND 5	28

A+ Education & AEI (Informit) search strategy

	SEARCH TERMS	A+
1 (P)	SUA="Allied health occupations education" OR SUA="Clinical teaching (Health professions)" OR ((health OR clinical OR clinician* OR medic* OR doctor* OR physician* OR nurse OR nursing OR pharmac* OR psycholog* OR dental OR dentist* OR midwi?e* OR physiotherap* OR "physical therap"* OR "occupational therap"* OR paramedic* OR radiotherapy OR radiation OR podiat* OR optometr* OR "speech therap"* OR "speech patholog"* OR dietetic OR dieti?ian* OR nutrition OR "exercise physiolog"* OR "exercise science"* OR chiropract* OR biomedic*) %2 student*) OR (("health profession"* OR "allied health" OR "health occupation"* OR "health science"* OR medic* OR clinical OR nurse OR nursing OR pharmacy OR psychology OR dental OR dentist* OR midwi?e* OR physiotherap* OR "physical therap"* OR "occupational therap"* OR paramedic* OR radiotherap* OR radiation OR podiatr* OR optometr* OR dietetic* OR dieti?ian* OR "speech therap"* OR "speech patholog"* OR chiropract* OR biomedic* OR "public health") %2 education)	5608
2 (I&C)	SUA="Learning activities" OR SUA="Lecture method" OR SUA="Computer assisted teaching" OR SUA="Distance education" OR SUA="Problem based learning" OR SUA="Case studies" OR SUA="Small group teaching" OR SUA="Workshops" OR SUA="Online learning" OR SUA="Independent study" OR SUA="Blended learning" OR SUA="Direct instruction" OR SUA="Experimental teaching" OR curricul* OR teaching OR learning OR lecture* OR "computer assisted instruction" OR "distance education" OR PBL OR CBL OR "case stud"* OR workshop* OR e-learning OR elearning OR "independent study" OR "independent studies" OR "direct instruction"*	137108
3 (O)	SUA="Evidence based practice" OR "evidence based practice"* OR EBP OR EBPC OR "evidence based medicine" OR EBM OR "evidence based nursing" OR EBN OR "evidence based healthcare" OR EBHC OR "evidence based dentistry" OR "evidence based decision"* OR "evidence informed practice" OR (best %2 evidence) OR SUA="Decision making" OR SUA="Decision making skills" OR SUA="Problem solving" OR SUA="Critical thinking" OR PICO OR "clinical question"* OR "literature search"* OR "critical appraisal" OR "critical analys"* OR "critical think"* OR "decision making" OR "problem solving"	13629
4	1 AND 2 AND 3	516
5	control* trial*	255
6	4 AND 5	6
